# Metabolomic Profiling of Dongxiang Wild Rice Under Salinity Demonstrates the Significant Role of Amino Acids in Rice Salt Stress

**DOI:** 10.3389/fpls.2021.729004

**Published:** 2021-09-22

**Authors:** Yaling Chen, Wenxue Huang, Fantao Zhang, Xiangdong Luo, Biaolin Hu, Jiankun Xie

**Affiliations:** ^1^Laboratory of Plant Genetic Improvement and Biotechnology, Jiangxi Normal University, Nanchang, China; ^2^Rice Research Institute, Jiangxi Academy of Agricultural Sciences, Rice National Engineering Laboratory, Nanchang, China

**Keywords:** salinity, dongxiang wild rice, metabolome, amino acid, L-asparagine

## Abstract

Dongxiang common wild rice is a precious rice germplasm resource for the study and improvement of salt tolerance in rice.The metabolism profile of Dongxiang wild rice (DXWR) under salinity was determined by high performance liquid chromatography-mass spectrometry (HPLC-MS) to find differential metabolites and screen potential biomarkers for salt-tolerant rice varieties. A global untargeted metabolism analysis showed 4,878 metabolites accumulated in seedlings of Dongxiang wild rice. Principal component analysis (PCA) and orthogonal partial least squares-discriminant analysis (OPLS-DA) results provided a clear metabolism discrimination between DXWR under control and DXWR under salinity. A total of 90 metabolites were significantly changed (49 upregulated and 41 downregulated) under salinity, of which the largest increase was in DL-2-Aminoadipic acid (27.08-fold) and the largest decrease was in L-Carnitine (0.014-fold). Amino acids and nuclear glycosides were mainly upregulated, while carbohydrates and organic acids were mainly downregulated in the salt-treated group. Among the top 10 upregulated metabolites, five kinds of differential metabolites were amino acids. According to the survival rates of the seedlings under salinity, we selected three backcross inbred lines of DXWR with survival rates above 80% as salt-tolerant progenies (pro-DS) and three backcross inbred lines with survival rates below 10% as non-salt-tolerant progenies (pro-NDS) for an amino acid change analysis. This analysis found that the change in L-Asparagine (2.59-fold) was the biggest between pro-DS and pro-NDS under salinity, revealing that the contents of L-Asparagine may be one of the indices we can use to evaluate the salt tolerance of rice varieties.

## Introduction

Rice is a crucial cereal crop in developing countries as it feeds more than half of the global population. However, soil salinity is one of the major environmental stresses that influences rice growth and development in many rice cultivation regions because rice is a glycophyte and, therefore, highly susceptible to salinity stress, especially at the seedling and reproductive stages (Huang et al., 2020; Zhang et al., 2021b). Furthermore, there are 1 × 10^9^ hectares of saline-alkali land worldwide, accounting for about 20% of total agricultural land (Munns and Tester, 2008; Liu et al., 2017). In addition, the salinization area is still expanding. It has been estimated that nearly 50% of all the cultivated land in the world will be salinized by 2050 (Zhu, 2016), posing a major threat to sustainable agricultural development and food security. Thus, the development and cultivation of salt-tolerant cultivated rice are some of the effective strategies to increase the utilization rate of salinity land, enlarge the area of arable soil, and ensure the national food supply.

As an ancestor of modern rice, cultivated rice species were mostly domesticated from common wild *Oryza rufipogon* species, but some abiotic stress-related traits were lost in the process of domestication. One particular variety that descended from this process was Dongxiang common wild rice, which was discovered in Dongxiang County (N 28°14'), Jiangxi, China, in 1978 (Tian et al., 2006; Xie et al., 2010) and is the northernmost region where any species of wild rice grows. This variety is a rich source of potential genes related to high grain yield, disease and insect resistance, cytoplasmic male sterility, fertility restoration, wide cross-compatibility, cold, and drought tolerance (Quan et al., 2018; Li et al., 2019; Qi et al., 2020; Wu et al., 2020). Zhou et al. (2016) also found that, compared with cultivated rice, Dongxiang wild rice had a very high survival rate under salt stress, suggesting that it is a precious plant germplasm resource for the genetic improvement of salt tolerance in cultivated rice.

Salt stress is the combined effect of osmotic and ion toxicity (primary effect) and oxidative stress (secondary effect). Plants respond to salt stress through many strategies, including the selective preferential uptake and exclusion of K+ ions over Na+ ions (Silveira et al., 2003; Hanin et al., 2016; Su et al., 2020) and the elimination of reactive oxygen species (ROS) through the antioxidant defense system (Tofighi et al., 2017; Wu et al., 2017). Moreover, the synthesis of small organic solutes such as proline, glycine, polyamines, glucose, inositol, glycerol, sugars, and betaine enhances the osmotic pressure of the plant cell against salinity (Latz et al., 2013; Nan et al., 2016).

Metabolomics plays a vital role in studying the changes of metabolites caused by external environment changes or genetic modification, which is the closest research field of the phenotype, and is also one of the hotspots in the field of genomic research. For instance, under drought, high temperature, and multiple salt stress, Sun et al. (2016) found significant changes in citric acid, malic acid, aspartic acid, glucose, fructose, proline, alanine, and threonine in maize leaves by NMR. Supporting this, Guo et al. (2017) systematically analyzed the changes in the metabolic groups of maize seedlings under salt stress by gas chromatography-mass spectrometry (GC-MS), which found that salt changed plant gluconeogenesis and inhibited photosynthesis, nitrogen metabolism, glycolysis, and the synthesis of many kinds of amino acids. Thus, the identification and quantitative study of plant tissue metabolomic changes under salt stress will provide insight into the salinity tolerance mechanisms of rice (Yaish and Kumar, 2015). Furthermore, the metabolomic profiling of salt-stressed rice from roots and leaves revealed that salt affected the xylem sap metabolic group and significantly decreased the concentrations of tricarboxylic acid (TCA) cycle intermediates and the shikimic acid pathway (Zuther et al., 2007). The study of different rice genotypes by Hakim et al. (2014) also showed that, with the increase of salt stress, the contents of proline and reducing sugars increased while the levels of chlorophyll content, non-reducing sugars, and grain yield decreased, implicating a high degree of genotypic variation in metabolomes under salt stress. Other previous studies have shown that salt increases ROS and some antioxidants in rice (Formentin et al., 2018; Jana and Yaish, 2020). Although technological progress has enabled the usage of various analytical techniques, such as gas chromatography–time-of-flight mass spectrometry (GC-TOF-MS), to study the global metabolites in specific stressed cells or tissues, few studies have clarified the metabonomic changes of *O. rufipogon* due to salt stress.

The discovery and identification of key metabolites play an essential role in the mechanism of salt tolerance. As such, the purpose of this study was to determine the mechanism of salt tolerance based on non-targeted metabonomic analysis. Therefore, in this study, ultra-high performance liquid chromatography-quadrupole time-of-flight mass spectrometry (UHPLC-Q-TOF-MS) and ultra-high performance-multiple reaction monitoring-mass spectrometry (UHPC-MRM-MS) analysis techniques were used to analyze and evaluate the functions of the differentially accumulated metabolites in the seedlings of Dongxiang wild rice and their introgressive lines under control and salt treatments. The results obtained from this study revealed that Dongxiang wild rice could accumulate a large number of metabolites in varying degrees under salinity, while a few of them played a potential role in the mechanism of salt tolerance.

## Materials and Methods

### Plant Materials

The seeds of Dongxiang common wild rice (*Oryza rufipogon* Griff.) DY80 were collected in an experimental paddy field at the Rice Research Institute, Jiangxi Academy of Agricultural Sciences, Jiangxi, China. Cultivated rice R974 (recurrent parent) and DY80 (donor parent) were used to produce the F1 hybrid. Then, the F1 hybrid was backcrossed to R974 to form a BC_1_F_1_ population. Finally, the BC_1_F_9_ population (backcross inbred lines, BILs) was obtained using the BC_1_F_1_ population for nine consecutive inbred times. Dongxiang common wild rice (DXWR) and BILs were used to study rice metabolism under salt stress.

Mature seeds were germinated in the dark on multiple filter papers moistened with double-distilled water in a Petri dish, and the uniformly germinated seeds were sown in 96-well plates supported by a plastic container. The seedlings were grown under photoperiodic conditions (light for 16 h and darkness for 8 h) at 28°C in a lightproof chamber, and the growing culture solution was updated two times a day. After 14 days of seedling growth, the culture solution was replaced with the same solution supplemented with 0 and 200 mM of NaCl, as previously described (Zhou et al., 2016). For the metabolomic analysis, 20 seedlings were collected and mixed to minimize the effect of metabolome unevenness among the plants. The experiment was designed with a random complete block and repeated six times.

### Metabonomic Analysis

#### Extraction of Tissue Metabolites

Plant tissues (60 mg) homogenized in liquid nitrogen were ultrasonically broken with 1 ml of a methanol/acetonitrile (2:2:1, v/v) solution at a 4°C temperature for 30 min (Villaret et al., 2020). The broken tissues were incubated for 1 h to precipitate protein, then centrifuged at 13,000 × g with a micro-centrifuge at 4°C for 10 min. Afterward, the supernatant was collected and dried in a speed vacuum concentrator. The process was then repeated six times for each sample (biological replicates).

#### UHPLC-Q-TOF MS Analysis

Rice extracts were analyzed using the Agilent 1290 ultra-high-performance liquid chromatography system (Agilent Technologies, California, USA) coupled with electrospray ionization quadrupole time-of-flight mass spectrometry system (AB SCIEX, Framingham, USA). The samples were separated by chromatography and randomly injected into an ACQUITY UPLC BEH column (2.1 × 100 mm, with a particle size of 1.7 μm) (Acquity, Waters, Milford, MA, USA). In the process of analysis, the sample tray was kept at 4°C. The injection volume was 2 μl and the column was kept at 25°C. Quality control (QC) samples are injected five times for analytical control.

The flow rate for the mobile phase was set at 0.3 ml/min throughout the gradient. For eluent A, 25 mM of ammonium acetate, 25 mM of ammonium hydroxide, and double-distilled water were used, while acetonitrile was used as eluent B. The following gradient profile was employed: 0–1 min, 95% B; 1–14 min, 95–65% B; 14–16 min, 65–40% B; 16–18 min, 40% B; 18–18.1 min, 40–95% B; 18.1–23 min, 95% B.

A dual electronically stored information source was operated in positive and negative ionization modes. The detailed mass spectrometry (MS) conditions were set according to the method of Julijana et al. (2013). For data acquisition, the time-of-flight mass spectrometry (TOF MS) scan at a mass-to-charge ratio of 60–1,000 Da was set at an accumulation time of 0.2 s/spectra, and product ion scan at a m/z of 25–1,000 Da was set at an accumulation time of 0.05 s/spectra. The secondary MS was performed by information-dependent acquisition (IDA). The IDA parameters were set as follows: exclude isotopes within 4 Da and have six candidate ions to monitor per cycle.

#### Data Processing and Multivariate Statistical Analysis

The raw data files were converted to a mass spectrometer output file format using the software tool MZConvert (ProteoWizard, proteowizard.sourceforge.net). Retention time alignment, peak integration, and peak alignment were then performed with XCMS software. Afterward, peaks were picked using the cenWave algorithm [(parameters: m/z = 25 ppm, prefilter = c (10, 100), peak width = c (10, 60)] and grouped using the Obiwarp algorithm (parameters: bw = 5, mzwid =.025, minfrac = 0.5) (Benton et al., 2015). The peak of the missing values >50% in the group were then removed from the data matrix. After data processing, a two-dimensional matrix was generated, which was composed of m/z and retention time (RT) data pairs, and the mass value and intensity of the peak were output to Excel for further chemometric analyses.

A multivariate statistical analysis of the dataset was carried out using the SIMCA-P software (version 14.1, UMETRICS, Umeå, Sweden) (Nicholson et al., 1999). A principal component analysis (PCA), which consists of score plots and loading plots, showed the contrast between the different samples, while the load chart explains the inherent changes in the data matrix. The identification markers of different rice classes were screened by an orthogonal partial least square discriminant analysis (OPLS-DA). According to the physical separation in the S-plot, the identified ions were determined manually and then used for the identification of potential biomarkers.

#### Pathway Enrichment and Clustering Analysis

To clarify the metabolic pathway of secondary metabolites, the compounds identified in the above databases were submitted to the Kyoto Encyclopedia of Genes and Genomes (KEGG) (http://www.genome.jp/kegg/pathway.html). A hierarchical clustering analysis (HCA) was performed by PermutMatrix (Caraux and Pinloche, 2005) using z-score transformed metabolite abundances and clustering on their Pearson distances (Minoru et al., 2012).

### Amino Acid Determination

#### Metabolite Extraction

The sample was ground into powder after freeze-drying. Then, 60 mg of each sample were accurately weighed and transferred to a centrifugal tube. After adding two steel balls and 1,000 μl extraction solvent (acetonitrile-methanol-water 2:2:1, including the mixture of internal standards of isotopically-labeled) pre-cooled at −20°C, the samples were mixed with a vortex, homogenized at 40 Hz for 4 min, and sonicated for 5 min in an ice-water bath. The homogenate and ultrasonic cycles were repeated three times and then incubated at −40°C for 1 h and centrifuged at 12,000 rpm at 4°C for 15 min (Mushtaq et al., 2014). Finally, the clear supernatant of 80 μl was transferred to an auto-sampler vial for UHPLC-tandem MS (MS/MS) analysis.

#### UHPLC-MRM-MS Analysis

Rice extracts were separated by chromatography and randomly injected into a Waters ACQUITY UPLC BEH amide column (100 × 2.1 mm, 1.7 μm) using an Agilent 1290 Infinity II series UHPLC System (Agilent Technologies). In the process of analysis, the sample tray temperature was kept at 4°C. The injection volume was 1 μl, while the column temperature was kept at 35°C. Mobile phase A was a 1% formic acid aqueous solution, while the mobile phase B was a 1% formic acid acetonitrile solution.

An Agilent 6460 triple quadrupole mass spectrometer (Agilent Technologies) equipped with a Jet Stream Technology Ion Source (AJS) electrospray ionization (Agilent Technologies, California, USA) (AJS-ESI) interface was applied for MS. The parameters of the ion source were set by Dunn et al. (2011).

#### Data Processing and Multivariate Statistical Analysis

The lower detection limits and lower limits of quantitation were determined by the signal-to-noise ratio. According to the US FDA guidelines for the validation of the bioanalytical method (Saccenti et al., 2014), lower detection limits (LLODs) and lower limits of quantitation (LLOQs) are defined as the analyte concentrations that lead to peaks of signal-to-noise ratio (S/N) at 3 and 10, respectively. In the sample, the content of the target metabolite was calculated according to the following formula.


$$c_{\text{M}}[nmol•g^{\;-\text{1}}]=\frac{C_{F}\;[nmol•L^{-1}]•V[mL]}{m[mg]}$$

Where C_F_ is the final concentration, C_M_ is the metabolite concentration, V is the final volume, and m is the quality of the sample.

## Results

### Metabolic Profiling of Salt-Stress Responses in Wild Rice

To investigate the influence of salt conditions on rice seedling metabolites, both rice seedlings were analyzed under non-salt and salt conditions using an ultra-high performance liquid chromatography-quadrupole time-of-flight mass spectrometry-based metabolomic approach. As shown in Supplementary Figure 1, there was a significant difference in the intensities of the compounds peaks in the total ion chromatography (TIC) plot obtained by the UHPLC-Q-TOF-MS analysis between the two samples, especially for peaks between the retention times of 4 and 12 min in the TIC plot (Supplementary Figure 1). According to the data treatment procedures, for an efficient comparison of the two organic rice samples under non-salt and salt conditions, 4,484 positively correlated variables were detected and 3,441 negatively correlated variables were detected (Supplementary Tables 1, 2).

### PCA and OPLS-DA

**Figure 1:**
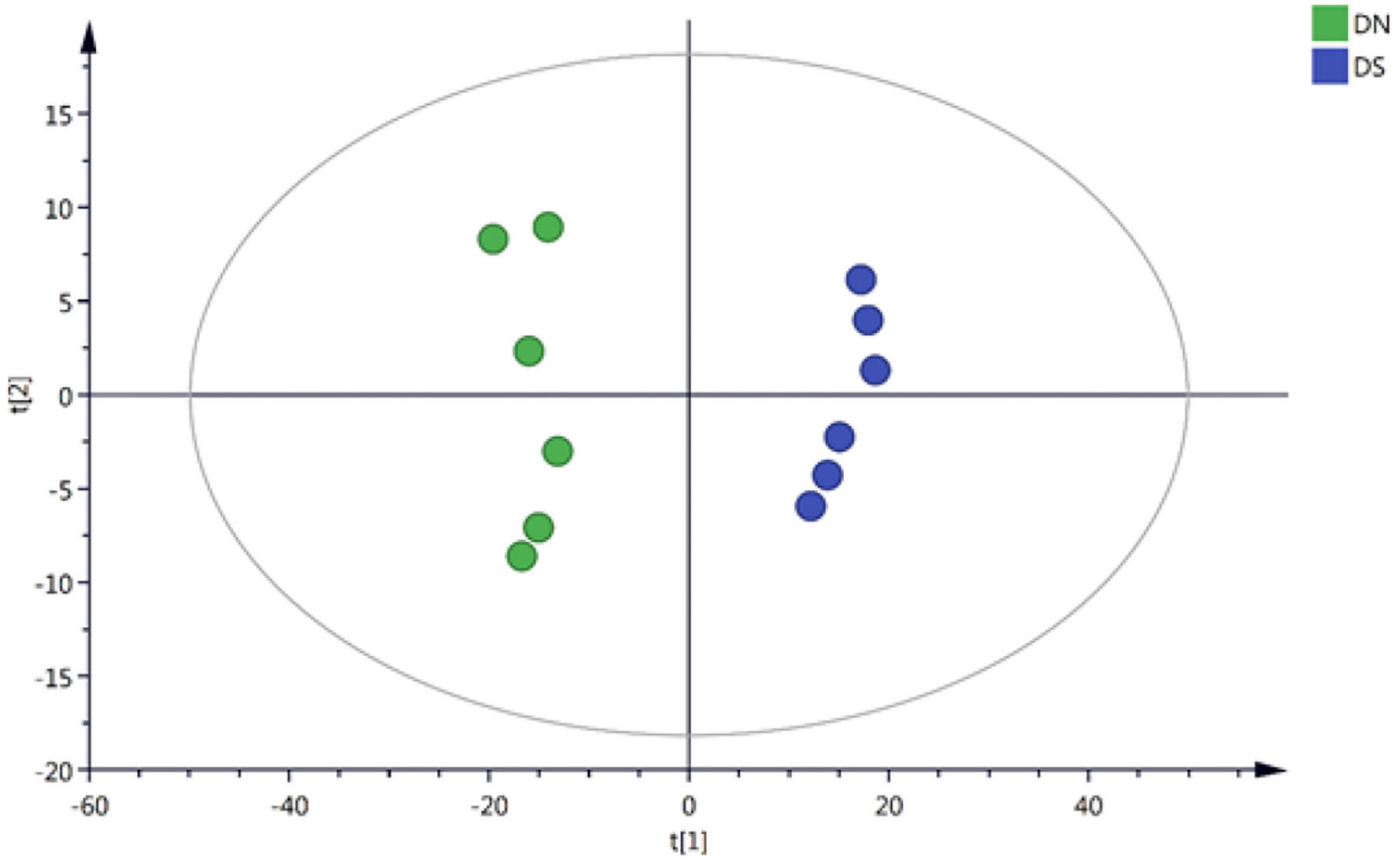
Principal component analysis (PCA) score plots for two samples of DN and DS. DN is Dongxiang wild rice (DXWR) without salt treatment and DS is DXWR under salinity.

The 4,484 positively correlated variables and 3,441 negatively correlated variables were normalized based on internal standards and intensities and used to perform multivariate statistical analyses. The quality of the model was evaluated by R2 and Q2 values. R2, which explains the proportion of variance, explained, and predicted in the PCA model separately. Specifically, 65.7 and 69.5% of the positively correlated variances (R2) and negatively correlated variances (R2), respectively, were explained by the first two components. On the other hand, the Q2 values were 45.2 and 51.5%, respectively (Figure 1), for the same variances. All these values showed the validity of the model. These results suggested that the, and DXWR under salinity (DS) and DXWR without salt treatment (DN) samples were distributed in different groups according to the first two principal components, while there were no outlier samples. This indicated that there were metabolic differences between the DN and DS groups.

To strengthen the distinction between the experimental groups and improve the validity and reliability of the results, an OPLS-DA was used to accurately evaluate the metabolic patterns of these samples. In Supplementary Figure 2, the OPLS-DA parameters of the model were expressed by the R2 of 0.834 and Q2 of –0.329 (Supplementary Figure 2). These parameters exhibited the better stability and predictability of the model and effectively reflected the metabolic differences between the DN and DS responses to salt stress.

### Hierarchical Cluster Analysis of Total Metabolites

**Figure 2:**
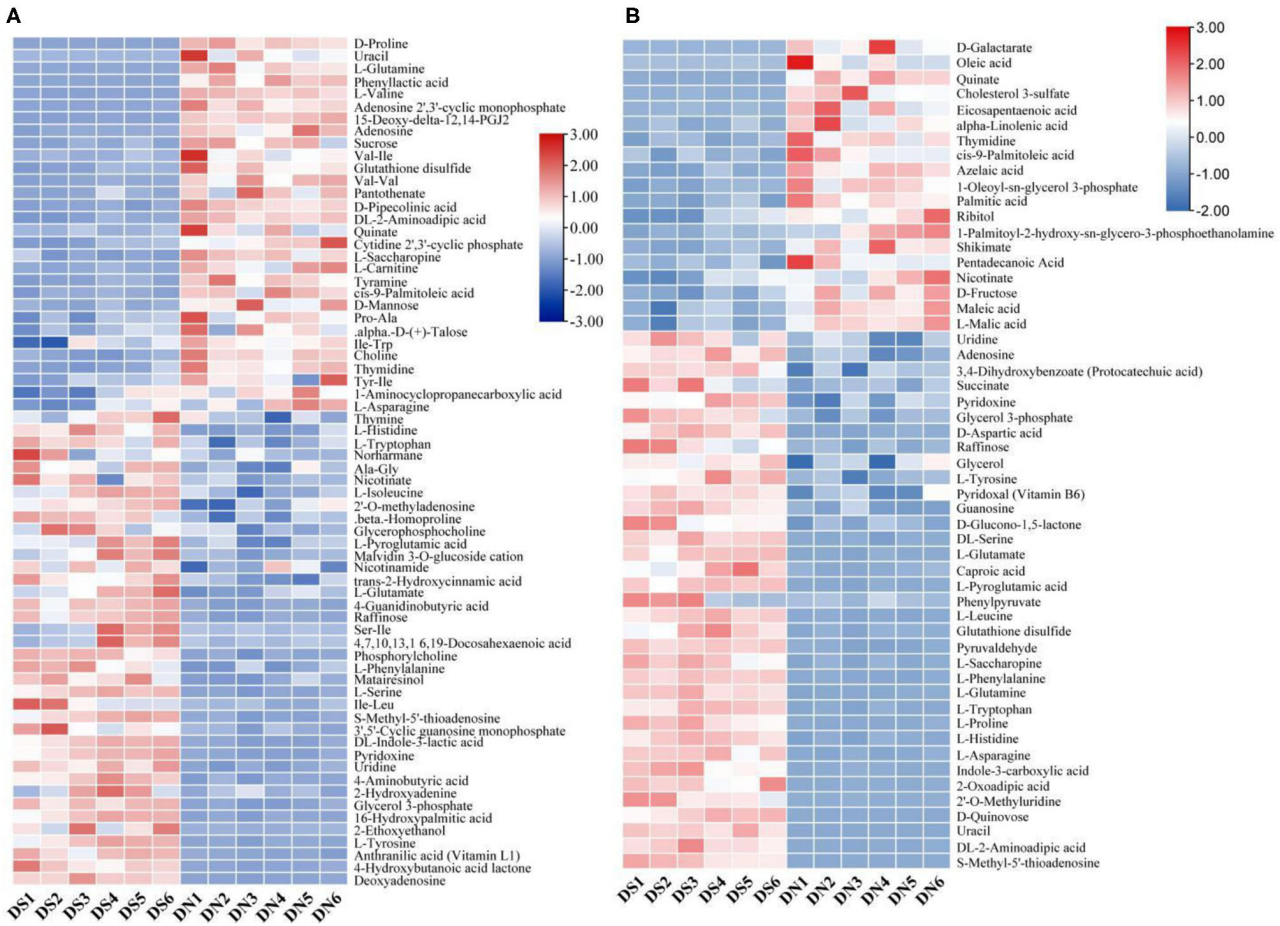
Cluster analysis of a positive ion model **(A)** and a negative ion model **(B)** in DN and DS. Red indicates a high expression of metabolites and blue indicates a low expression of metabolites. Columns represent all samples and rows represent all metabolites. DN is DXWR without salt treatment and DS is DXWR under salinity.

By clustering all the identified metabolites, the metabolites of six samples in the DS group were clustered into one group (Figure 2), indicating that the metabolic patterns of the samples were similar after salt treatment. At the same time, the DN and DS groups formed relatively clear high and low expression regions, while the high and low expression regions of the DN group were opposite to those of the DS group, indicating that salt stress had a significant effect on rice metabolism.

### Differential Metabolite Analysis in Rice Under Salt Stress

**Figure 3:**
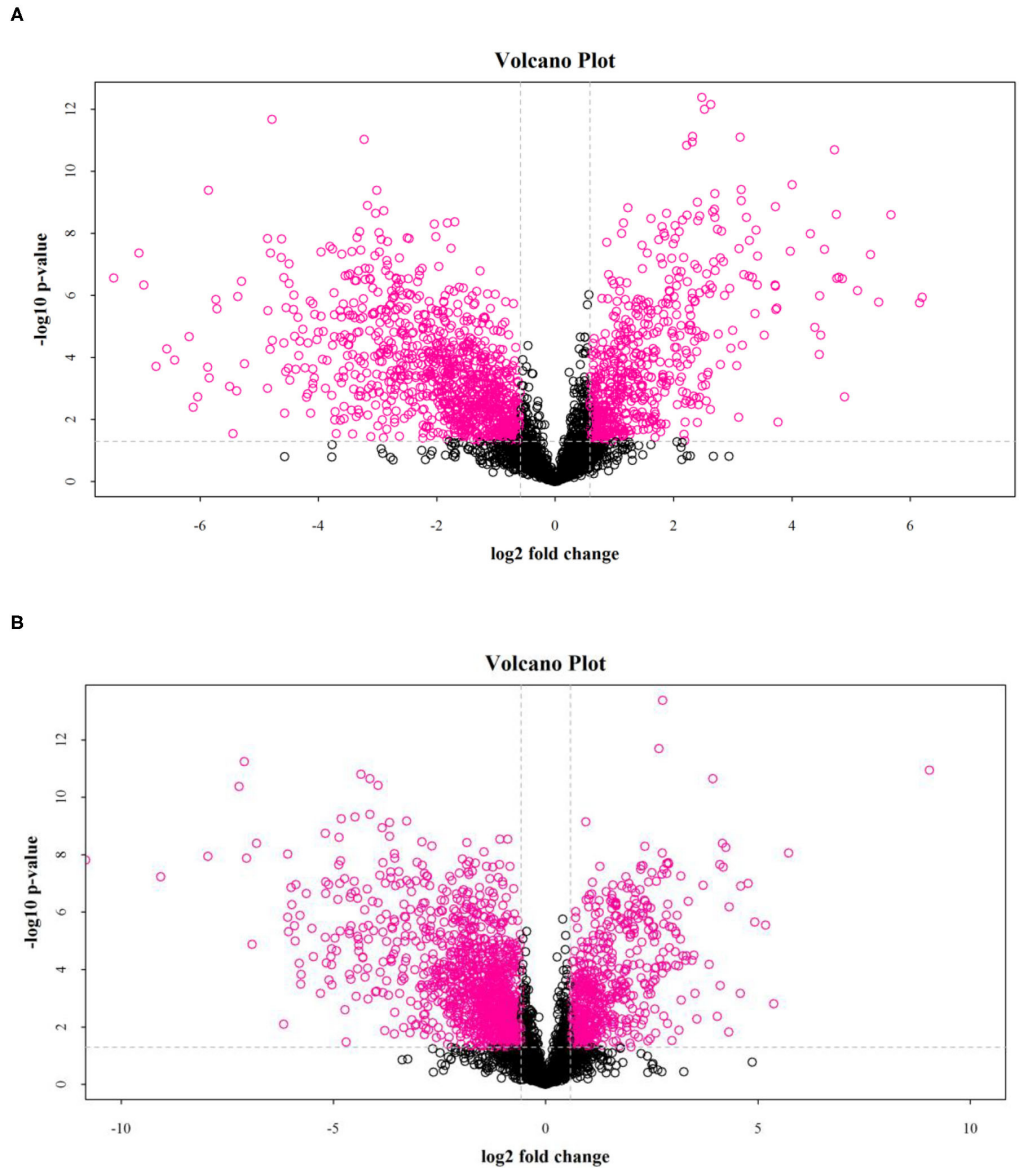
Volcanic diagrams of a positive ion model **(A)** and a negative ion model **(B)** between DN and DS. Each point in the picture represents a metabolite. DN is DXWR without salt treatment and DS is DXWR under salinity.

The results showed that salt stress significantly affected the distribution of metabolites in rice. As shown in Figure 3, the volcano map of differential metabolites (fold change > 2 and *p* < 0.05) showed significant differences between the DN and DS groups.

Using a variable importance in the projection screening >1 at a level of *p* < 0.05, a total of 90 differential metabolites were isolated in the positive ion mode and negative ion mode. Compared with DN, 49 differential metabolites in DS were upregulated and 41 were downregulated. The 10 differential metabolites with the most extensive upregulated range were DL-2-Aminoadipic acid (27.08-fold), Uracil (26.89-fold), D-Quinovose (23.38-fold), 2'-O-Methyluridine (22.4-fold), 2-Oxoadipic acid (13.438), Indole-3-carboxylic acid (13.26-fold), L-Asparagine (10.67-fold), L-Proline (9.12-fold), L-Histidine (10.49-fold), L-Tryptophan (6.881-fold), and L-Glutamine (6.32-fold). The most downregulated differential metabolite was L-Carnitine (0.014-fold).

**Figure 4:**
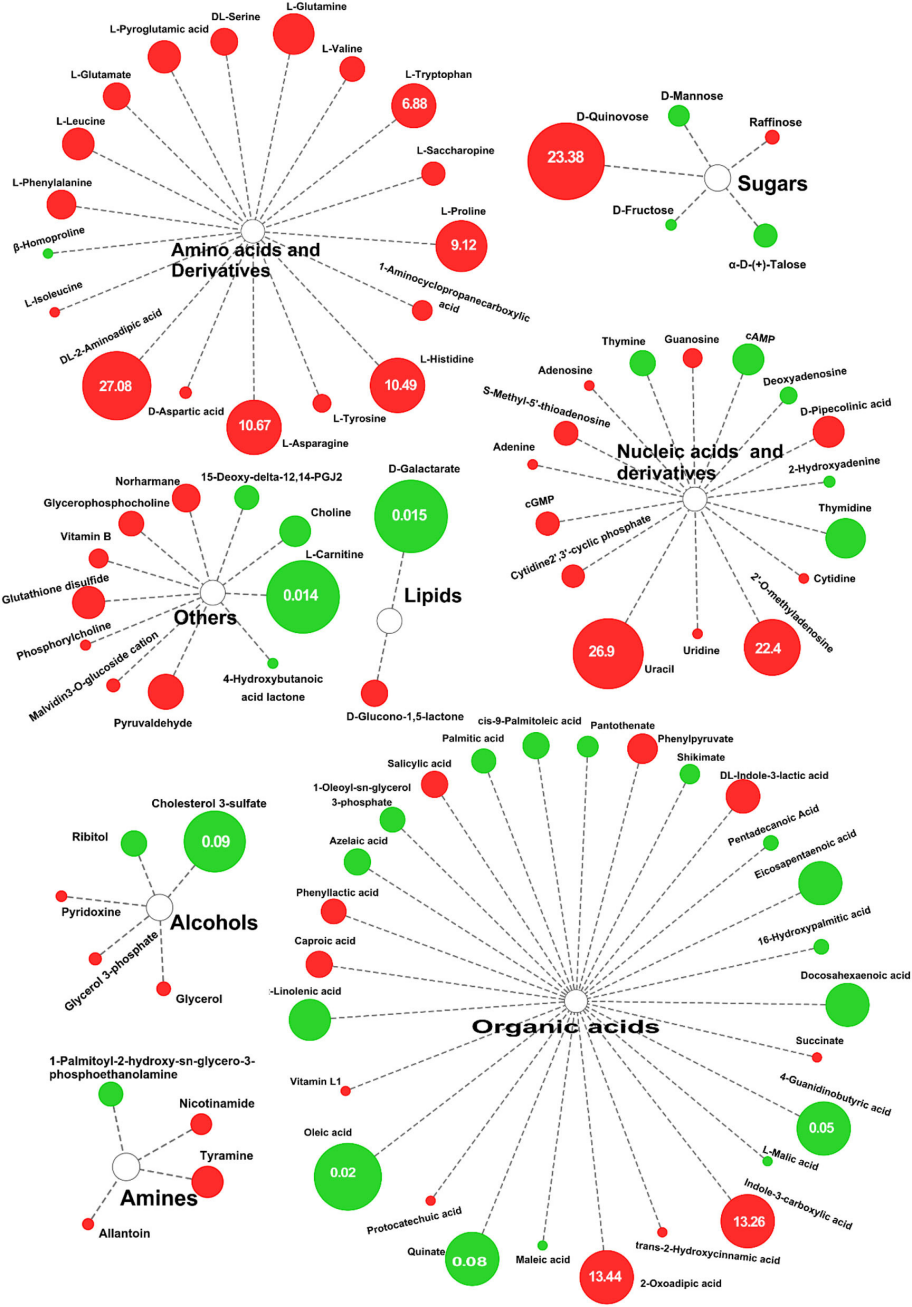
Differential metabolites of DN and DS. The sizes of the circles reflect FC in abundance between DN and DS; red indicates significantly upregulated in DS, green indicates significantly downregulated. DN is DXWR without salt treatment and DS is DXWR under salinity.

According to Figure 4, there were 88 differential metabolic species, including 18 amino acids and their derivatives (all are up-regulated), 27 organic acids and their derivatives, 16 nucleosides and their metabolites, 5 sugars, 5 alcohols, 4 organic amines, 2 lipids, and 11 other substances. Similarly, in the negative ion mode, a total of 54 differential metabolites were screened. Compared with the control group, there were 35 upregulated metabolites in DS, including 15 amino acids and their derivatives, of which S-Methyl-5'-thioadenosine (40.107) was the most upregulated (Supplementary Table 3). There were 19 downregulated metabolites, of which the largest decrease was in D-Galactarate (0.015) (Supplementary Table 3). From the differences in the types and expression of different metabolites, salt stress had the greatest effect on the amino acid metabolism of rice.

### KEGG Pathway Analysis for Differential Metabolites

**Figure 5:**
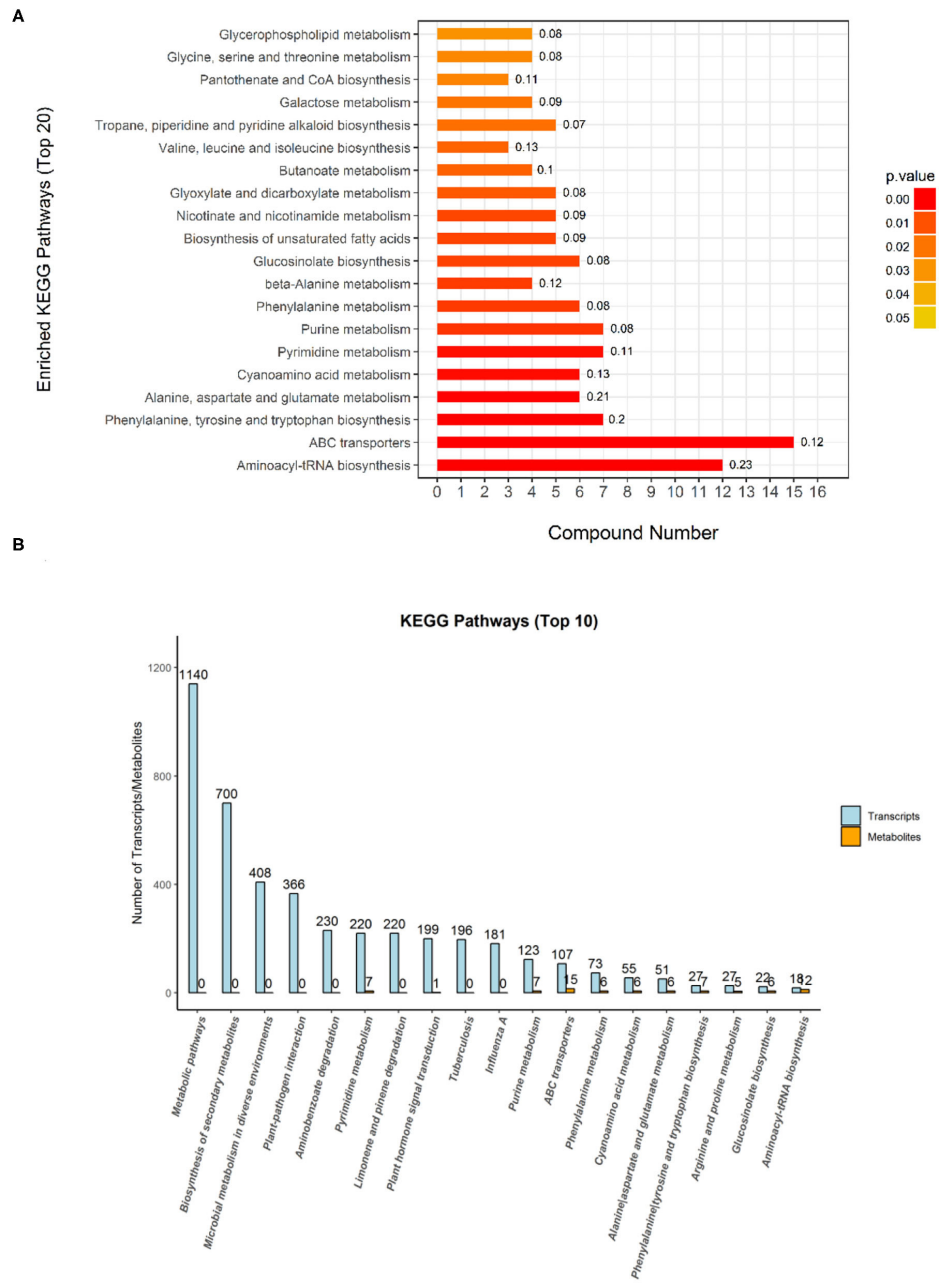
The Kyoto Encyclopedia of Genes and Genomes (KEGG) classification of the differential metabolites **(A)** and transcriptomes **(B)** between DN and DS. The metabolic pathways annotated in KEGG are classified according to the type of pathway. The Abscissa is the number of metabolites annotated to that pathway and the ratio of the number of metabolites annotated to the total number of annotated metabolites in the corresponding pathway. DN is DXWR without salt treatment and DS is DXWR under salinity.

The Kyoto Encyclopedia of Genes and Genomes is one of the databases commonly used in communication research, as researchers use it to read a large amount of literature to describe many metabolic pathways and their relationships to a specific graphical language. In this study, a KEGG analysis was used to annotate differential metabolites. Compared with the metabolites of the DN group, the DL-2-Aminoadipic acid with the largest upregulation of the metabolites in the DS group was annotated in the amino acid biosynthesis and metabolic pathway. As shown in Figure 5, the KEGG pathway enrichment analysis of the differentially expressed metabolites of DN/DS compared with the control group by Fisher precise test showed that significant changes had taken place in important pathways such as Aminoacyl-tRNA biosynthesis, ABC transporters, Alanine, Aspartate and Glutamate metabolism, Purine metabolism, and Galactose metabolism. Among the top 20 KEGG pathways, 9 were related to amino acid synthesis. Next, a conjoint analysis of the metabolome and transcriptome was performed (Zhou et al., 2016), which found that 6 pathways (251 genes) were involved in amino acid metabolism among top the 10 KEGG pathways. The results indicated that amino acid metabolism could be used as a physiological indicator to evaluate rice salt tolerance.

### Morphological Traits in the Progenies of Dongxiang Wild Rice Under Salinity

**Figure 6:**
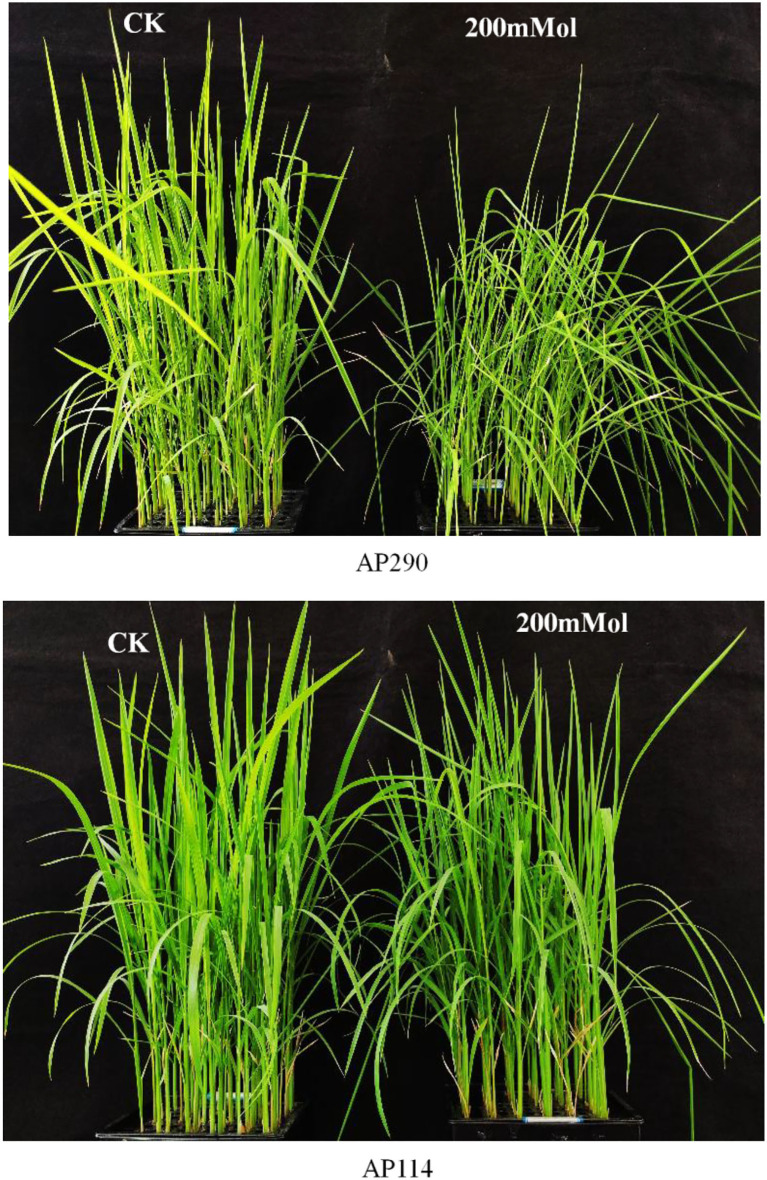
Comparison of phenotypes between saline-treated plants and blank controls (CK).

According to previous studies, more than 80% of Dongxiang wild rice under seedlings survived under a 200-mM NaCl treatment for 12 days, which is a much higher amount than what common cultivated rice can survive (Zhou et al., 2016). To study the changes in the metabolites in the introgression lines of DXWR, 2-week-old seedlings of 130 BILs were exposed to a hydroponic solution with 200 mM of NaCl for 12 days. Under normal growth conditions, the 130 BIL seedlings survived and retained their green color. Under salt stress conditions, however, most BIL leaves became curled up, wilted, and lost their green color. Thereafter, the seedlings were returned to normal growth conditions, resulting in most of BILs not being able to restore normal growth and only a small number of BILs resuming growth. According to the survival rates of the seedlings under salinity, 3 BILs with survival rates above 80% were selected as AP17, AP92, and AP114 and 3 BILs with survival rates below 10% as AP91, AP290, and AP10, respectively, for a differential metabolite analysis (Figure 6). Additionally, AP17, AP92, and AP114 were defined as salt-tolerant progenies (pro-DS), whereas AP91, AP290, and AP10 were non-salt-tolerant (pro-NDS).

### Changes of Amino Acid Content in the Progenies of Dongxiang Wild Rice

According to the metabonomic analysis, salt stress significantly affected the amino acid metabolism of Dongxiang wild rice. In order to study the changes in the amino acids in the progenies of Dongxiang common wild rice under salt stress, Student's *t*-test (*p* < 1) was used to screen the differential metabolites between salt-tolerant and non-salt-tolerant progenies under salinity (Table 1). In the two groups, the five amino acids with concentrations more than 4,000 (nmol/g) were the same, namely, L-Serine, L-Glutamic acid, L-Alanine, L-Glutamine, and L-Aspartic acid. Moreover, the three amino acids with the lowest expression were the same, namely, 4-Hydroxyproline, 3-Methyl-L-histidine, and 1-Methyl-L-histidine. The change in L-Asparagine was the biggest between pro-DS and pro-NDS under salinity, while the fold change of pro-DS/pro-NDS was 2.5-fold. Moreover, the contents of L-Aspartic acid (1.44-fold) and L-Glutamine (1.77-fold) were also upregulated in pro-DS. On the contrary, Alanine, Methyl-L-histidine, and L-Citrulline were downregulated in pro-DS. Thus, the changes in amino acid content in the progenies were consistent with the metabonomic characteristics of DXWR.

**Table 1:** Contents of amino acid in rice under salt stress.

**Compound name**	**Pro-DS**			**Pro-NDS**			**FC(Pro-DS/Pro-NDS)**
	**AP114**	**AP17**	**AP92**	**AP191**	**AP290**	**AP10**	
Glycine	1,040.34 ± 2.20	847.86 ± 4.58	1,229.21 ± 9.37	892.23 ± 7.69	941.27 ± 8.39	890.81 ± 3.72	1.14
L-Alanine	4,639.10 ± 7.20	4,690.11 ± 9.01	4,785.14 ± 7.37	5,399.67 ± 12.69	5,750.66 ± 9.39	5,345.59 ± 9.35	0.86
4-Aminobutyric acid	2,691.36 ± 4.20	3,045.38 ± 4.20	2,601.45 ± 6.37	2,279.43 ± 9.69	2,091.21 ± 8.39	2,161.10 ± 11.35	1.28
L-Serine	6,248.31 ± 9.20	6,484.08 ± 6.89	6,443.23 ± 3.63	6,530.61 ± 12.69	6,683.32 ± 9.39	6,739.96 ± 6.35	0.96
L-Proline	260.52 ± 5.20	261.73 ± 6.31	274.73 ± 3.37	205.03 ± 5.69	208.00 ± 7.39	218.57 ± 8.35	1.26
L-Valine	1,628.49 ± 10.2	2,306.62 ± 6.41	1,835.07 ± 5.37	1,833.83 ± 5.69	1,652.26 ± 9.39	1,772.85 ± 8.35	1.10
L-Threonine	1,922.85 ± 7.80	2,265.98 ± 4.11	2,175.50 ± 3.20	1,858.04 ± 8.69	1,889.41 ± 4.45	1,749.09 ± 9.35	1.16
4-Hydroxyproline	20.42 ± 4.20	20.81 ± 2.51	20.19 ± 2.38	18.19 ± 2.69	18.25 ± 4.39	18.49 ± 3.35	1.12
L-Ornithine	32.46 ± 4.20	33.98 ± 2.71	36.32 ± 2.37	30.42 ± 1.69	33.15 ± 4.59	27.12 ± 4.35	1.13
L-Asparagine	5,681.20 ± 5.80	4,620.06 ± 6.31	4,360.77 ± 7.19	1,826.07 ± 9.69	1,773.24 ± 8.39	2,065.20 ± 6.86	2.59
L-Aspartic acid	6,628.70 ± 12.20	7,054.69 ± 7.41	6,947.67 ± 6.37	4,631.25 ± 6.69	4,758.25 ± 14.39	4,941.49 ± 8.35	1.44
L-Glutamine	7,849.36 ± 49.80	8,070.76 ± 6.61	7,056.67 ± 9.37	4,245.5 ± 6.69	4,426.77 ± 12.39	4,276.76 ± 7.35	1.77
L-Lysine	829.01 ± 12.40	840.31 ± 6.91	799.48 ± 10.37	807.76 ± 5.69	834.77 ± 8.39	856.41 ± 10.35	0.99
L-Glutamic acid	23,722.51 ± 25.8	20,033.05 ± 10.31	22,058.94 ± 13.37	15,734.36 ± 7.69	17,763.61 ± 9.89	17,617.24 ± 10.35	1.29
L-Methionine	179.86 ± 6.20	195.22 ± 3.61	183.53 ± 7.37	155.54 ± 3.67	145.38 ± 7.39	139.74 ± 5.35	1.27
L-Histidine	288.13 ± 4.90	316.45 ± 4.81	277.72 ± 10.37	218.06 ± 5.69	247.45 ± 9.39	265.13 ± 8.35	1.21
L-Phenylalanine	310.93 ± 8.20	339.75 ± 1.41	305.06 ± 7.37	391.65 ± 4.69	371.75 ± 7.39	370.64 ± 8.35	0.84
3-Methyl-L-histidine	5.62 ± 1.40	5.45 ± 1.61	5.21 ± 1.83	6.24 ± 2.69	6.65 ± 3.39	6.63 ± 2.35	0.83
1-Methyl-L-histidine	7.07 ± 1.40	7.14 ± 2.61	7.74 ± 1.37	10.39 ± 1.69	10.76 ± 2.39	10.01 ± 2.35	0.70
L-Arginine	407.42 ± 8.1	475.49 ± 4.39	398.60 ± 9.38	416.12 ± 6.69	414.73 ± 7.39	432.42 ± 10.35	1.01
L-Citrulline	138.74 ± 6.80	143.26 ± 5.39	144.06 ± 5.37	230.12 ± 5.69	241.06 ± 7.39	251.10 ± 5.91	0.59
L-Tyrosine	286.13 ± 10.77	301.42 ± 6.39	246.28 ± 6.37	277.84 ± 5.69	256.69 ± 9.39	239.12 ± 5.35	1.08
L-Tryptophan	150.20 ± 6.24	202.04 ± 6.41	167.92 ± 7.37	166.57 ± 5.69	143.48 ± 7.61	165.20 ± 6.35	1.09

*Pro-DS was salt-tolerant progenies under salt stress and pro-NDS was non-salt-tolerant progenies under salt stress*.

*FC, fold change*.

## Discussion

In rice salinity phenotyping related to the seedling, the major visible phenotypic symptoms include plant survival rates, phenotypic expression (yellowing tip burning, browning, and rolling in younger leaves), and poor root growth. Salt stress also influences numerous biochemical processes, i.e., chlorophyll content, CO2 emission, malondialdehyde (MDA), and superoxide dismutase (SOD) (Hussain et al., 2019). The symptoms of salinity have also been often found to overlap with those of other abiotic stresses. Therefore, there is, unfortunately, no single clear-cut trait or criterion in rice salinity phenotyping. Previous studies have shown that physiological and biochemical processes and more than 6,800 genes of DXWR were changed under salt stress (Zhou et al., 2016; Ai et al., 2020), while the overexpression of miR1861h from DXWR in particular increased tolerance to salt stress in cultivated rice (Ai et al., 2020). In this study, a total of 68 metabolites were significantly changed under salinity. Most of the upregulated metabolites were nuclear glycosides and amino acids. Carbohydrates and organic acids, in contrast were mainly downregulated. Furthermore, 15 differential metabolites significantly or extremely significantly affected 20 metabolic pathways such as phenylalanine, tyrosine, and tryptophan biosynthesis, alanine-aspartic acid-glutamic acid metabolism, and the TCA cycle. Of these significant pathways, nine were amino acid metabolic pathways, indicating that amino acid metabolism can be used as a physiological indicator to evaluate rice salt tolerance.

The accumulation of osmotic protective agents is very important to the alleviation of the intracellular osmotic imbalance caused by salt stress in plants. Proline, in particular, is an important osmotic regulator that maintains the osmotic balance between the protoplast and the environment. Previous studies have shown that salt stress significantly increased levels of proline accumulation (Forlani et al., 2019; Gerona et al., 2019). Specifically, the fold changes of D-Proline and L-Proline were 7.893 and 9.124 in DXWR under salinity, respectively. Hussain et al. (2019) also found that proline contents were increased in two cultivated rice varieties under salt stress. Additionally, Ganie et al. (2019) revealed that the activity of the proline biosynthesis gene *OsP5CS* was inhibited by the feedback of proline content and had a significant effect on rice resistance to salt stress. Furthermore, glutamic acid, the substrate for the synthesis of proline, was also upregulated, a finding that was consistent with the findings of a study by Zhang et al. (2021a) on the metabonomics of salt-tolerant rice varieties in Changbai.

The content of basic and neutral amino acids was increased in Dongxiang wild rice under salinity. Moreover, S-Methyl-5'-thioadenosine (40.107) was the most upregulated in negative ion differential metabolites. Wang et al. (2019) found that more basic (Lys, Arg) and neutral (Val, Ala) amino acids were transported and accumulated in the amino acid permease 5 (OsAAP5) overexpression lines than in the wild type. Additionally, OsAAP5 could regulate tiller bud outgrowth by affecting zeatin levels. Zeatin is ubiquitous in most bioassays with extremely high activity and is considered to play an important role in plant growth and development (Mok and Mok, 2001). Adenosine and S-Methyl-5'-thioadenosine are also synthetic precursors of trans zeatin formed by adenosine phosphate-isopentenyltransferase (IPT). There are seven IPT genes in Arabidopsis (*Arabidopsis thaliana*), of which IPT3 is upregulated by nitrate (Sakakibara, 2006). The absorption and assimilation of nitrate by plants are usually closely combined with nitrogen (N) metabolism. Nitric oxide, produced as part of N metabolism, is one of the most widely used signal molecules in organisms. Furthermore, polyamines and nitric oxide are related to the salt tolerance of plants (Ahmad et al., 2016; Khushboo and Gyan, 2019).

Asparagine is an important amino acid for the long-distance transport of nitrogen in plants. Degenkolbe et al. (2013) reported that higher levels of asparagine (Asn) were predominantly found in drought-sensitive cultivars under drought stress. However, little is known about the effect of Asn on rice salt tolerance. In this study, the leaves of DXWR and pro-DS presented slighter wilting than pro-NDS after salt stress. The contents of L-Asparagine were also increased 10.675-fold in DXWR under salt stress. More interestingly, the change in L-Asparagine (2.59-fold) was the biggest between pro-DS and pro-NDS. Asparagine has a high concentration of C/N and is more stable compared with other amide compounds. Furthermore, two asparagine synthase genes were identified in rice, with one *OsASN1* located on chromosome 3 and the other annotated as *OsASN2* located on chromosome 6. The analysis of the T-DNA insertion mutant showed that *OsASN1* is involved in the regulation of rice development and is specific for tiller outgrowth (Luo et al., 2019). In a previous study, *OsASN1* and cyanoalanine synthetase were upregulated under salt stress in DXWR seedlings (Zhou et al., 2016). Cyanoalanine synthetase in salt-tolerant rice catalyzes the conversion of cyanide to cyanoalanine, which is further transformed into asparagine and enters the amino acid pool of rice. This may also be one of the reasons why, among the top ten upregulated metabolites, five kinds of differential metabolites were amino acids. Therefore, the contents of L-Asparagine may be one of the indices to evaluate the salt tolerance of rice varieties. However, the related speculations need to be further studied and verified.

## Data Availability Statement

The original contributions presented in the study are included in the article/Supplementary Material, further inquiries can be directed to the corresponding authors.

## Author Contributions

YC and WH: investigation, methodology, formal analysis, and writing of the original draft. FZ: methodology and supervision. XL: resources and methodology. BH: conceptualization, resources, methodology, writing, reviewing and editing, supervision, and project administration. JX: conceptualization, resources, methodology, writing, review and editing, supervision, and project administration. All authors contributed to the article and approved the submitted version.

## Funding

This research was supported by the Natural Science Foundation of China (Grant Nos. 31760378 and 31800640) awarded to BH and YC. The major projects of the key research programs in Jiangxi Province (Grant No. 20192ACB60013) were awarded to JX. The Natural Science Foundation of Jiangxi Province (Grant No. 2020BABL205018) was awarded to BH.

## Conflict of Interest

The authors declare that the research was conducted in the absence of any commercial or financial relationships that could be construed as a potential conflict of interest.

## Publisher's Note

All claims expressed in this article are solely those of the authors and do not necessarily represent those of their affiliated organizations, or those of the publisher, the editors and the reviewers. Any product that may be evaluated in this article, or claim that may be made by its manufacturer, is not guaranteed or endorsed by the publisher.

## References

[B1] Ahmad P. Abdel L. A. A. Hashem A. Abd A. E. F. Gucel S. Tran L. P. (2016). Nitric oxide mitigates salt stress by regulating levels of osmolytes and antioxidant enzymes in chickpea. Front. Plant Sci. 7, 347–357. 10.3389/fpls.2016.00347 27066020 PMC4814448

[B2] Ai B. Chen Y. Zhao M. Ding G. Xie J. K. Zhang F. T. (2020). Overexpression of miR1861h increases tolerance to salt stress in rice (*Oryza sativa* L.). Genet. Resour. Crop Ev. 68, 87–92. 10.1007/s10722-020-01045-9

[B3] Benton H. P. Ivanisevic J. Mahieu N. G. Kurczy M. E. Johnson C. H. Franco L.. (2015). Autonomous metabolomics for rapid metabolite identification in global profiling. Anal. Chem. 87, 884–891. 10.1021/ac5025649 25496351 PMC4303330

[B4] Caraux G. Pinloche S. (2005). PermutMatrix: a graphical environment to arrange gene expression profiles in optimal linear order. Bioinformatics 21, 1280–1281. 10.1093/bioinformatics/bti141 15546938

[B5] Degenkolbe T. Do P. T. Kopka J. Zuther E. Hincha D. K. Kohl K. I. (2013). Identification of drought tolerance markers in a diverse population of rice cultivars by expression and metabolite profiling. PLoS ONE 8:e63637. 10.1371/journal.pone.0063637 23717458 PMC3661581

[B6] Dunn W. B. Broadhurst D. Begley P. Zelena E. Francis M. S. Anderson N.. (2011). Procedures for large-scale metabolic profiling of serum and plasma using gas chromatography and liquid chromatography coupled to mass spectrometry. Nat. Protoc. 6, 1060–1083. 10.1038/nprot.2011.335 21720319

[B7] Forlani G. Bertazzini M. Cagnano G. (2019). Stress-driven increase in proline levels, and not proline levels themselves, correlates with the ability to withstand excess salt in a group of 17 Italian rice genotypes. Plant Biol. 21, 336–342. 10.1111/plb.12916 30253007

[B8] Formentin E. Sudiro C. Ronci M. B. Locato V. Barizza E. Stevanato P.. (2018). H_2_O_2_ signature and innate antioxidative profile make the difference between sensitivity and tolerance to salt in rice cells. Front. Plant Sci. 9:1549. 10.3389/fpls.2018.01549 30405678 PMC6206305

[B9] Ganie S. A. Molla K. A. Henry R. J. Bhat K. V. Mondal T. K. (2019). Advances in understanding salt tolerance in rice. Theor. Appl. Genet. 132, 851–870. 10.1007/s00122-019-03301-8 30759266

[B10] Gerona M. E. B. Deocampo M. P. Egdane J. A. Ismail A. M. Dionisio-Sese M. L. (2019). Physiological responses of contrasting rice genotypes to salt stress at reproductive stage. Rice Sci. 26, 207–219. 10.1016/j.rsci.2019.05.001

[B11] Guo R. Shi L. Yan C. Zhong X. Gu F. Liu Q. (2017). Ionomic and metabolic responses to neutral salt or alkaline salt stresses in maize (*Zea mays* L.) seedlings. BMC Plant Biol. 17, 41–53. 10.1186/s12870-017-0994-6 28187710 PMC5301417

[B12] Hakim M. Juraimi A. S. Hanafi M. Ismail M. R. Selamat A. Rafii M. (2014). Biochemical and anatomical changes and yield reduction in rice (*Oryza sativa* L.) under varied salinity regimes. Biomed Res. Int. 2014:208584. 10.1155/2014/208584 24579076 PMC3919121

[B13] Hanin M. Ebel C. Ngom M. Laplaze L. Masmoudi K. (2016). New insights on plant salt tolerance mechanisms and their potential use for breeding. Front. Recent Dev. Plant Sci. 7:1787. 10.3389/fpls.2016.01787 27965692 PMC5126725

[B14] Huang L. Wu D. Zhang G. (2020). Advances in studies on ion transporters involved in salt tolerance and breeding crop cultivars with high salt tolerance. J. Zhejiang Univ. Sci. B. 21, 426–441. 10.1631/jzus.B1900510 32478490 PMC7306632

[B15] Hussain S. Bai Z. Huang J. Cao X. Zhu L. Zhu C.. (2019). 1-Methylcyclopropene modulates physiological, biochemical, and antioxidant responses of rice to different salt stress levels. Front. Plant Sci. 10:124. 10.3389/fpls.2019.00124 30846992 PMC6393328

[B16] Jana G. A. Yaish M. W. (2020). Functional characterization of the Glyoxalase-I (*PdGLX1*) gene family in date palm under abiotic stresses. Plant Signal. Behav. 15:1811527. 10.1080/15592324.2020.1811527 32835595 PMC7588186

[B17] Julijana I. Zhu Z. J. Lars P. Ralf T. Stephen C. O'Brien P. J.. (2013). Toward 'omic scale metabolite profiling: a dual separation-mass spectrometry approach for coverage of lipid and central carbon metabolism. Anal. Chem. 85, 6876–6884. 10.1021/ac401140h 23781873 PMC3761963

[B18] Khushboo K. Gyan S. S. (2019). Nitric oxide improved salt stress tolerance by osmolyte accumulation and activation of antioxidant defense system in seedling of *B. juncea* (L.). Czern. Vegetos. 32, 583–592. 10.1007/s42535-019-00071-y

[B19] Latz A. Mehlmer N. Zapf S. Mueller T. D. Wurzinger B. Pfister B. (2013). Salt stress triggers phosphorylation of the arabidopsis vacuolar K + channel TPK1 by calcium-dependent protein kinases (CDPKs). Mol. Plant. 6, 1274–1289. 10.1093/mp/sss158 23253603 PMC3971370

[B20] Li L. Chen H. Mao D. (2019). Pyramiding of rapid germination loci from *Oryza sativa* cultivar 'xieqingzao b' and cold tolerance loci from Dongxiang wild rice to increase climate resilience of cultivated rice. Mol. Breed. 39, 85. 10.1007/s11032-019-0985-4

[B21] Liu Y. Wang B. Li J. Song Z. Lu B. Chi M.. (2017). Salt-response analysis in two rice cultivars at seedling stage. Acta Physiol. Plant. 39, 215. 10.1007/s11738-017-2514-6 31736527 PMC6858053

[B22] Luo L. Qin R. Liu T. Yu M. Yang T. Xu G. (2019). OsASN1 plays a critical role in asparagine-dependent rice development. Int. J. Mol. Sci. 20, 130–144. 10.3390/ijms20010130 30602689 PMC6337572

[B23] Minoru K. Susumu G. Yoko S. Miho F. Mao T. (2012). KEGG for integration and interpretation of large-scale molecular data sets. Nucleic Acids Res. 40, D109–D114. 10.1093/nar/gkr988 22080510 PMC3245020

[B24] Mok D. W. Mok M. C. (2001). Cytokinin metabolism and action. Annu. Rev. Plant Physiol. Plant Mol. Biol. 52, 89–118. 10.1146/annurev.arplant.52.1.89 11337393

[B25] Munns R. Tester M. (2008). Mechanisms of salinity tolerance. Annu. Rev. Plant Biol. 59, 651–681. 10.1146/annurev.arplant.59.032607.092911 18444910

[B26] Mushtaq M. Y. Choi Y. H. Verpoorte R. Wilson E. G. (2014). Extraction for metabolomics: access to the metabolome. Phytochem. Anal. 25, 291–306. 10.1002/pca.2505 24523261

[B27] Nan G. Zhang Y. Li S. Lee I. Takano T. Liu S. (2016). NaCl stress-induced transcriptomics analysis of *Salix linearistipularis* (syn. *Salix mongolica*). J. Biol. Res. 23:1. 10.1186/s40709-016-0038-7 26933650 PMC4772304

[B28] Nicholson J. K. Lindon J. C. Holmes E. (1999). 'Metabonomics': understanding the metabolic responses of living systems to pathophysiological stimuli via multivariate statistical analysis of biological NMR spectroscopic data. Xenobiotica 29, 1181–1189. 10.1080/004982599238047 10598751

[B29] Qi W. Chen H. Yang Z. Hu B. Luo X. Ai B.. (2020). Systematic characterization of long non-coding RNAs and their responses to drought stress in Dongxiang wild rice. Rice. Sci. 27, 21–31. 10.1016/j.rsci.2019.12.003

[B30] Quan R. Wang J. Hui J. Bai H. Lyu X. Zhu Y.. (2018). Improvement of salt tolerance using wild rice genes. Front. Plant Sci. 8:2269. 10.3389/fpls.2017.02269 29387076 PMC5776132

[B31] Saccenti E. Hoefsloot H. C. Smilde A. K. Johan A. (2014). Reflections on univariate and multivariate analysis of metabolomics data. Metabolomics 10, 361–374. 10.1007/s11306-013-0598-6

[B32] Sakakibara H. (2006). Cytokinins: activity, biosynthesis, and translocation. Annu. Rev. Plant Biol. 57, 431–449. 10.1146/annurev.arplant.57.032905.105231 16669769

[B33] Silveira J. A. Viégas R. A. Rocha I. M. Moreira A. C. Moreira R. A. Oliveira J. T. (2003). Proline accumulation and glutamine synthetase activity are increased by salt-induced proteolysis in cashew leaves. J. Plant Physiol. 160, 115–123. 10.1078/0176-1617-00890 12685027

[B34] Su Q. Zheng X. Tian Y. Wang C. (2020). Exogenous brassinolide alleviates salt stress in *Malus hupehensis* Rehd. by regulating the transcription of NHX-Type Na+(K+)/H+ antiporters. Front. Plant Sci. 11:38. 10.3389/fpls.2020.00038 32117377 PMC7016215

[B35] Sun C. Li M. Gao X. Liu L. Wu X. Zhou J. (2016). Metabolic response of maize plants to multi-factorial abiotic stresses. Plant Biol. 18, 120–129. 10.1111/plb.12305 25622534

[B36] Tian F. Li D. Fu Q. Zhu Z. Fu Y. Wang X.. (2006). Construction of introgression lines carrying wild rice (*Oryza rufipogon* Griff.) segments in cultivated rice (*Oryza sativa* L.) background and characterization of introgressed segments associated with yield-related traits. Theor. Appl. Genet. 112, 570–580. 10.1007/s00122-005-0165-2 16331476

[B37] Tofighi C. Khavari-Nejad R. A. Najafi F. Razavi K. Rejali F. (2017). Responses of wheat plants to interactions of 24-epibrassinolide and *Glomus mosseae* in saline condition. Physiol. Mol. Biol. Plants 23, 557–564. 10.1007/s12298-017-0439-6 28878494 PMC5567700

[B38] Villaret C. J. Poupin N. Tournadre A. Aurélie B. Bertrand M. J. (2020). An optimized dual extraction method for the simultaneous and accurate analysis of polar metabolites and lipids carried out on single biological samples. Metabolites 10, 338–355. 10.3390/metabo10090338 32825089 PMC7570216

[B39] Wang J. Wu B. Lu K. Wei Q. Qian J. Chen Y.. (2019). The amino acid permease 5 (OsAAP5) regulates tiller number and grain yield in rice. Plant Physiol. 180, 1031–1045. 10.1104/pp.19.00034 30890663 PMC6548276

[B40] Wu T. Li X. Huang D. Huang F. Xiao Y. Hu B. (2020). Using Dongxiang wild rice backcross recombinant inbred lines to analyze QTLs related to low nitrogen tolerance and yield traits. Chin. J. Rice Sci. 34, 499–511. 10.16819/j.1001-7216.2020.0408

[B41] Wu W. Zhang Q. Ervin E. H. Yang Z. Zhang X. (2017). Physiological mechanism of enhancing salt stress tolerance of Perennial Ryegrass by 24-epibrassinolide. Front. Plant Sci. 8:1017. 10.3389/fpls.2017.01017 28674542 PMC5474491

[B42] Xie J. Hu B. Wan Y. Zhang T. Li X. Liu R.. (2010). Comparison of the drought resistance characters at seedling stage between Dongxiang common wild rice (*Oryza rufipogon* Griff.) and cultivars (*Oryza sativa* L.). Acta Ecol. Sin. 30, 1665–1674.

[B43] Yaish M. W. Kumar P. P. (2015). Salt tolerance research in date palm tree (*Phoenix dactylifera* L.), past, present, and future perspectives. Front. Plant Sci. 6, 348–352. 10.3389/fpls.2015.00348 26042137 PMC4434913

[B44] Zhang G. Zhu J. Sun M. Yan G. Liu K.. (2021a). Analysis of differential metabolites in Changbai No. 10 rice grains under salt stress. J. Integr. Agric. 54, 675–683. 10.3864/j.issn.0578-1752.2021.04.001

[B45] Zhang X. Wang X. Jia W. Xu Z. Wang Y. Wu L. (2021b). Research progress of plants under salt treatment. North. Hortic. 6, 137–143. 10.11937/bfyy.20201076

[B46] Zhou Y. Yang P. Cui F. Zhang F. Luo X. Xie J. (2016). Transcriptome analysis of salt stress responsiveness in the seedlings of Dongxiang wild rice (*Oryza rufipogon* Griff.). PLoS ONE 11:146–242. 10.1371/journal.pone.0146242 26752408 PMC4709063

[B47] Zhu J. K. (2016). Abiotic stress signaling and responses in plants. Cell 167, 313–324. 10.1016/j.cell.2016.08.029 27716505 PMC5104190

[B48] Zuther E. Koehl K. Kopka J. (2007). Comparative Metabolome Analysis of the Salt Response in Breeding Cultivars of Rice. Advances in molecular breeding toward drought and salt tolerant crops, Springer Netherlands.

