# Supplementary material for: Metabolomic Profiling of Dongxiang Wild Rice Under Salinity Demonstrates the Significant Role of Amino Acids in Rice Salt Stress
Source: Front Plant Sci. 2021 Sep 22;12:729004. doi: 10.3389/fpls.2021.729004 (PMC8494129; doi:10.3389/fpls.2021.729004)
Supplement: Supplementary file 1 [file Image_1.pdf]

A

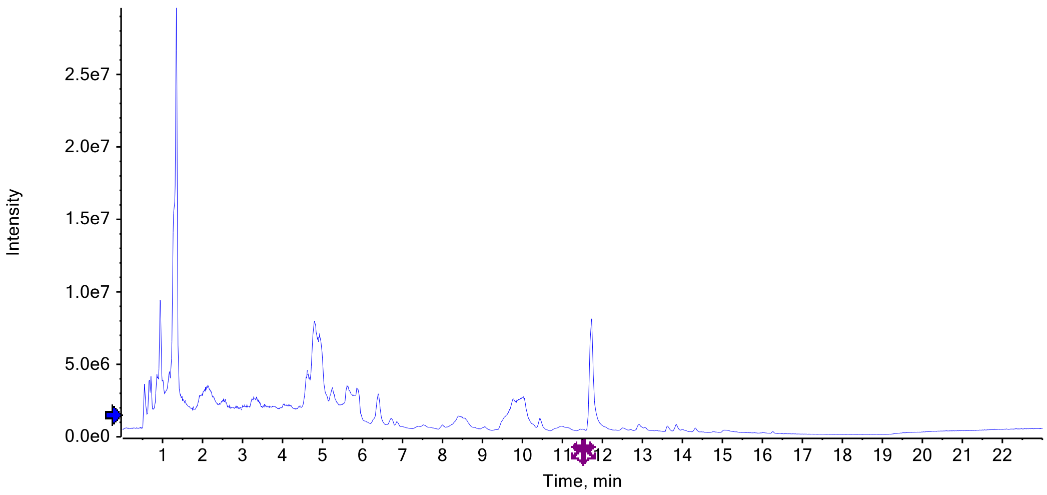

B

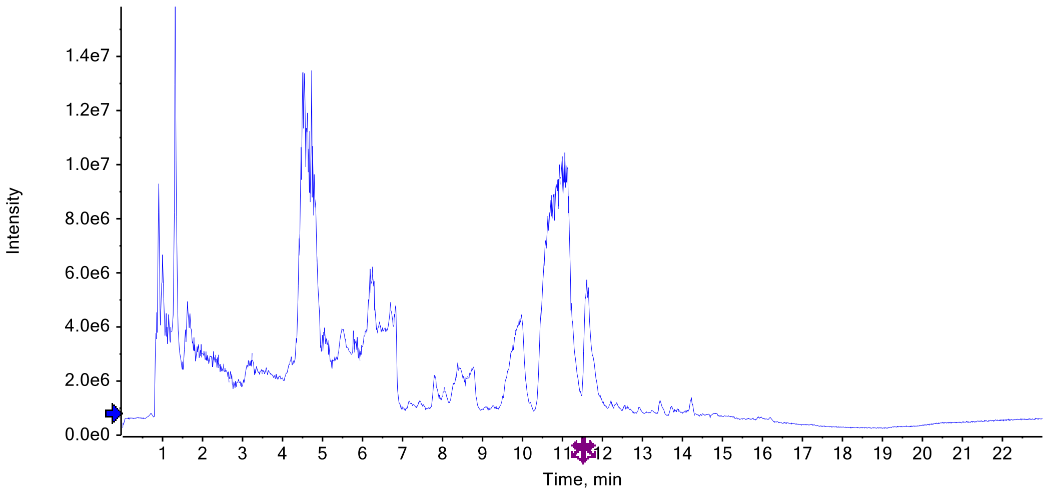

FIGURE S1 The total ion chromatogram (TIC) of metabolites determined by UHPLC-Q-TOF-MS in DN (A) and DS (B) rice. DN is rice without salt treatment, and DS is salt-tolerant rice under salt stress.
