# Supplementary material for: Metabolomic Profiling of Dongxiang Wild Rice Under Salinity Demonstrates the Significant Role of Amino Acids in Rice Salt Stress
Source: Front Plant Sci. 2021 Sep 22;12:729004. doi: 10.3389/fpls.2021.729004 (PMC8494129; doi:10.3389/fpls.2021.729004)
Supplement: Supplementary file 2 [file Image_2.pdf]

A

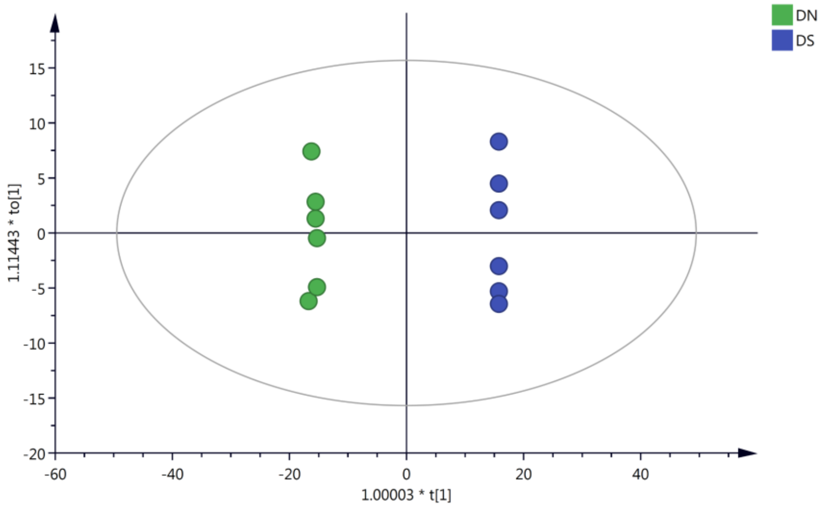

B

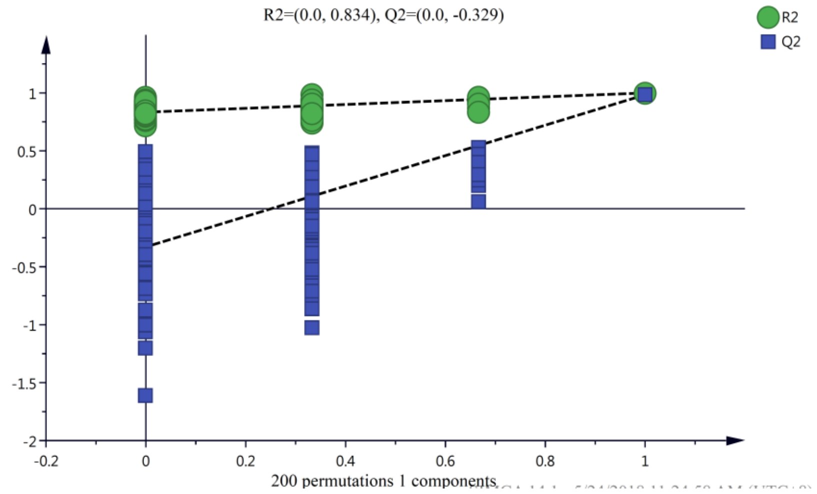

FIGURE S2 Orthogonal partial lease squares discriminant analysis (OPLS-DA) and permutation testing for Rice leaves. OPLS-DA score plots between DN and DS samples. (A). Permutation testing of the OPLS-DA models of DN and DS samples (B). DN is rice without salt treatment, and DS is salt-tolerant rice under salt stress.
