# Supplementary material for: Metabolomic Profiling of Dongxiang Wild Rice Under Salinity Demonstrates the Significant Role of Amino Acids in Rice Salt Stress
Source: Front Plant Sci. 2021 Sep 22;12:729004. doi: 10.3389/fpls.2021.729004 (PMC8494129; doi:10.3389/fpls.2021.729004)
Supplement: Supplementary file 5 [file Table_3.docx]

**TABLE 3** Differential metabolites of DN and DS in negative ion mode

| Category | Metabolic | Fold change | Significance |
| --- | --- | --- | --- |
|  |  | (DS/DN radio) |  |
| Amino acids and Derivatives | L-Phenylalanine | 5.729 | *** |
|  | L-Glutamine | 6.315 | *** |
|  | L-Leucine | 4.688 | *** |
|  | DL-Serine | 4.069 | *** |
|  | L-Histidine | 10.490 | *** |
|  | L-Tryptophan | 7.018 | *** |
|  | L-Pyroglutamic acid | 4.276 | *** |
|  | L-Glutamate | 4.077 | *** |
|  | D-Aspartic acid | 1.825 | *** |
|  | L-Asparagine | 10.675 | *** |
|  | L-Proline | 9.124 | *** |
|  | DL-2-Aminoadipic acid | 27.082 | *** |
|  | L-Saccharopine | 5.107 | *** |
|  | L-Tyrosine | 2.716 | *** |
| Organic acids | Quinate | 0.075 | *** |
|  | 2-Oxoadipic acid | 13.438 | *** |
|  | Indole-3-carboxylic acid | 13.262 | *** |
|  | Azelaic acid | 0.253 | *** |
|  | Palmitic acid | 0.270 | *** |
|  | 1-Oleoyl-sn-glycerol 3-phosphate | 0.260 | *** |
|  | L-Malic acid | 0.726 | *** |
|  | Caproic acid | 4.149 | *** |
|  | Shikimate | 0.329 | *** |
|  | Maleic acid | 0.714 | *** |
|  | 3,4-Dihydroxybenzoate (Protocatechuic acid) | 1.563 | *** |
|  | Eicosapentaenoic acid | 0.128 | ** |
|  | alpha-Linolenic acid | 0.151 | ** |
|  | cis-9-Palmitoleic acid | 0.238 | ** |
|  | Pentadecanoic Acid | 0.428 | ** |
|  | Succinate | 1.587 | ** |
|  | Nicotinate | 0.528 | * |
|  | Oleic acid | 0.238 | ** |
|  | Phenylpyruvate | 4.551 | * |
|  | Salicylic acid | 4.160 | * |
| Nucleic acids | S-Methyl-5'-thioadenosine | 40.107 | *** |
|  | Guanosine | 2.800 | *** |
|  | 2'-O-Methyluridine | 22.404 | *** |
|  | Adenosine | 40.107 | *** |
|  | Thymidine | 0.224 | *** |
|  | Uridine | 1.251 | ** |
|  | Cytidine | 1.272 | - |
| Sugars | D-Quinovose | 23.382 | *** |
|  | D-Fructose | 0.607 | *** |
|  | Raffinose | 1.940 | *** |
| Alcohols | Glycerol 3-phosphate | 1.776 | *** |
|  | Cholesterol 3-sulfate | 0.090 | *** |
|  | Pyridoxine | 1.644 | *** |
|  | Ribitol | 0.290 | ** |
|  | Glycerol | 1.985 | * |
| Amines | 1-Palmitoyl-2-hydroxy-sn-glycero-3-phosphoethanolamine | 0.316 | ** |
|  | Allantoin | 1.583 | - |
| Lipids | D-Glucono-1,5-lactone | 3.796 | *** |
|  | D-Galactarate | 0.015 | ** |
| Others | Pyruvaldehyde | 4.968 | *** |
|  | Uracil | 26.897 | *** |
|  | Glutathione disulfide | 4.886 | *** |
|  | Pyridoxal (Vitamin B6) | 2.773 | *** |
|  | Adenine | 1.187 | - |

Note: DN is rice without salt treatment, and DS is salt-tolerant rice under salt stress. – P>0.05; * 0.01<P<0.05; ** 0.001<P<0.01 and *** P<0.001.
